# Effect of Hypothyroidism on the Risk of Carpal Tunnel Syndrome and Electrodiagnostic Parameters

**DOI:** 10.3390/neurolint17090150

**Published:** 2025-09-18

**Authors:** Ahmad R. Abuzinadah

**Affiliations:** 1 Department of Neurology, Faculty of Medicine, King Abdulaziz University, Jeddah 21589, Saudi Arabia; aabuzinadah@kau.edu.sa; 2 Neuromuscular Medicine Unit, King Abdulaziz University Hospital, King Abdulaziz University, Jeddah 21589, Saudi Arabia; 3 Department of Internal Medicine, Neurology Division, International Medical Center, Jeddah 23214, Saudi Arabia

**Keywords:** carpal tunnel syndrome, hypothyroidism nerve conduction study, electrodiagnostic parameters, comparative study

## Abstract

Background: Hypothyroidism has been implicated as a risk factor for carpal tunnel syndrome (CTS). However, the effect of hypothyroidism on the risk of CTS has not been studied in large, non-selective clinic populations, and the impact of hypothyroidism on electrodiagnostic parameters remains inadequately understood. Methods: In this retrospective study, we examined 480 patients referred for upper limb electrodiagnostic evaluation. We compared the prevalence of CTS among patients with and without hypothyroidism, adjusting for age and gender. Additionally, we compared the median nerve sensory and motor latencies and comparative latency studies (COLS) [median-to-ulnar comparison through palmar difference (Palmdiff) and ring difference studies (Ringdiff); and median-to-radial comparison through a thumb difference study (Thumbdiff)] among patients with and without hypothyroidism disease, stratified by CTS status and age groups. Results: The crude prevalence of CTS was higher among patients with hypothyroidism (79.7%) compared to those without (61.8%) (*p* = 0.005). However, after adjusting for age and gender, logistic regression analysis revealed a non-significant association between hypothyroidism and CTS (adjusted odds ratio (OR): 1.71; 95% CI: 0.89–3.28, *p* = 0.106). CTS was more prevalent among patients with hypothyroidism under 50 years of age (OR: 2.59; 95% CI: 1.17–5.73, *p* = 0.018). There were no significant differences in any electrodiagnostic parameters between patients with and without hypothyroidism among CTS and non-CTS groups. Conclusions: Hypothyroidism increased the risk of CTS among patients under 50 years of age. The electrodiagnostic parameters used for CTS diagnosis were not influenced by the presence of hypothyroidism.

## 1. Introduction

Carpal tunnel syndrome (CTS) is an entrapment neuropathy, characterized by compression of the median nerve at the wrist. Thyroid dysfunction, particularly hypothyroidism, has been proposed as a risk factor for CTS [1,2]. The reported risk of CTS among hypothyroidism patients ranges from 7% to 92% [3,4,5,6]. This large variability in reported risk can be attributed to different study designs and populations as well as the CTS case definition. Some studies only included patients who underwent CTS decompression as CTS cases, while other studies used clinical and electrodiagnostic features to define CTS cases [4]. Proposed mechanisms for the increased risk of CTS with hypothyroidism include myxedematous infiltration, mucopolysaccharide deposition within the carpal tunnel, and resultant nerve ischemia or entrapment [7]. Moreover, given that the median nerve is connected to the surrounding fascia, hypothyroidism may cause thickening and swelling of the synovial membranes around the tendons [1,8]. Hypothyroidism was found to increase the risk of CTS by a odds ratio of 1.04 in two studies that used Mendelian randomization analysis [5,9]. This method of studying the association is less influenced by confounding variables; hence, it indicates a stronger association.

Several observational studies have reported an elevated prevalence of CTS in hypothyroid patients [1]. However, there are limitations in the current available literature, including the following: first, the risk of CTS among patients with hypothyroidism has typically been compared to a preselected control group or volunteer as opposed to a comprehensive clinic population that includes all patients presenting with hands symptoms who might have CTS; second, many previous studies did not exclude patient with diabetes, and the presence of DM influences the risk of CTS and the electrodiagnostic parameters [10]; third, it is unclear whether hypothyroidism influences the electrodiagnostic parameters used to confirm CTS, such as median nerve sensory and motor latencies or comparative latency studies. Electrodiagnostic testing remains a standard for CTS diagnosis and stratification, and understanding whether hypothyroidism affects electrodiagnostic features could have implications for diagnosis and management.

This study aimed to evaluate whether hypothyroidism is associated with an increased risk of carpal tunnel syndrome (CTS), both before and after adjusting for age and gender, and to examine whether hypothyroidism influences electrodiagnostic parameters across CTS and non-CTS groups stratified by age.

## 2. Materials and Methods

### 2.1. Study Design and Participants

This retrospective study used data that were systematically collected through structured patient interviews and documented in clinical records from January 2017 to December 2023 at King Abdulaziz University Hospital (KAUH) and the International Medical Center (IMC). The study protocol was approved by the institutional review boards of both centers. Participants were identified from the clinical neurophysiology laboratory records, including all patients referred for hand symptoms, to look for CTS as a possible etiology. Participants were eligible if they met the following criteria: (1) patients aged between 14 and 80 years; (2) patients presenting with upper limb paresthesia or pain; and (3) patients with documentation of presence or absence of hypothyroidism. Exclusion criteria included the following: (1) presence of diabetes mellitus (DM); and (2) presence of weakness proximal to the hands as part of the presenting complaint; this is because the presence of proximal weakness, such as deltoid, biceps, or triceps weakness, will exclude CTS from the differential diagnosis. Proximal pain is commonly reported with CTS; hence, it was not part of the exclusion criteria [11]. All eligible patients who met the inclusion criteria during the study period were included, representing a convenience sample.

### 2.2. Variables Definitions

A diagnosis of carpal tunnel syndrome (CTS) was made when at least two of the following three clinical features were present: (1) nighttime paresthesia; (2) worsening of symptoms during hand activities, such as driving, cycling, or phone use; and (3) reduction in the symptoms with hand shaking, commonly referred to as a positive flick sign [12,13]. It is widely accepted that clinical criteria can be used as the case definition in studies that investigate CTS diagnostic tests [14,15]. Thyroid disease status was determined from clinical records. All patients included in this study were directly asked whether they had been diagnosed with hypothyroidism or not, and this was documented in the electrodiagnostic report. We described the electrodiagnostic severity of CTS according to the classifications of Padua and Bland [16,17].

The structured interview included the following components: (1) nighttime paresthesia; (2) worsening of symptoms during hand activities, such as driving, cycling, or phone use; (3) reduction in the symptoms with hand shaking; (4) the site of maximum symptom; (5) presence of DM; and (6) whether the patient had been diagnosed with hypothyroidism. 

Patients were categorized into the following groups: (1) patients without CTS, which was subdivided into (a) patients without hypothyroidism and (b) patients with hypothyroidism; and (2) patient with CTS, which was subdivided into (a) patients without hypothyroidism and (b) patients with hypothyroidism.

### 2.3. Electrodiagnostic Data

A detailed description of the acquisition of the electrodiagnostic parameters was provided in our previous study [10].

### 2.4. Outcomes 

The primary objective of this study was to report the following among patients who were referred to the clinical neurophysiology lab with complaints of upper limb pain or paresthesia: (1) the crude prevalence of CTS among patients with and without hypothyroidism; (2) the comparison of the crude prevalence of CTS between patients with and without hypothyroidism using logistic regression; (3) the prevalence of CTS among patients with and without hypothyroidism, adjusted for age and gender; (4) the comparison of the adjusted prevalence of CTS between patients with and without hypothyroidism using logistic regression.

The secondary objective was to compare the following electrodiagnostic parameters between patients with and without hypothyroidism across both CTS and non-CTS groups: (1) median sensory nerve latency; (2) median motor nerve latency; (3) median versus ulnar latency difference through a digit IV/ring finger study (Ringdiff); (4) median versus ulnar latency difference through a mixed palmar study (Palmdiff); and (5) median versus radial latency difference through a digit I/thumb study (Thumbdiff). We used peak latency for sensory studies and onset latency for motor studies [18].

### 2.5. Statistical Analysis 

Continuous variables were described as median interquartile ranges (IQRs) and compared using the Mann–Whitney U test. Categorical variables were compared with the chi-square test. Prevalence was compared using logistic regression-calculated odds ratios (ORs) for uncontrolled comparison. Adjusted ORs were derived from a logistic regression analysis where CTS is the dependent variable, and hypothyroidism, age, and gender were all included in a single model as categorical independent variables. Adjusted prevalence values are predictive margins derived from logistic regression analysis. Given our sample sizes (69 patients with hypothyroidism and 411 patients without hypothyroidism), and the observed CTS prevalence (79.7% vs. 61.8%), our study has an estimated statistical power of approximately 85% at an alpha level of 0.05. Analyses were stratified by age categories (<50, 50–59, ≥60 years) and gender. Statistical analyses were performed using STATA version 13 (Stata-Corp, College Station, TX, USA).

## 3. Results

### 3.1. Patient Characteristics (Table 1)

The neurophysiology lab review revealed 672 patients who were referred for hand symptoms and suspicion for CT. A total of 181 patients were excluded as they have DM, while 11 additional patients were excluded due to insufficient data. We included 480 patients, of whom 309 (64.4%) had CTS. Thyroid disease was more prevalent among patients with CTS (17.8%) compared to those without CTS (8.2%) (*p* < 0.004). Among both groups (CTS and non-CTS), there were no differences between patients with and without hypothyroidism with respect to age distribution or disease duration. Males were more prevalent among the patients without CTS with no hypothyroidism. The durations of CTS symptoms were similar between patients with and without hypothyroidism. Additionally, the electrodiagnostic severity of CTS did not differ between patients with and without hypothyroidism.

### 3.2. Prevalence of CTS (Table 2)

The crude prevalence of CTS among patients with hypothyroidism was significantly higher (79.7%) than among those without hypothyroidism (61.8%) (OR: 2.42; 95% CI: 1.31–4.51, *p* = 0.005). After adjusting for age and gender, there was no difference in the prevalence of CTS (adjusted OR: 1.71; 95% CI: 0.89–3.28, *p* = 0.106). When comparing CTS prevalence across different age groups, there were more CTS cases among hypothyroidism patients in the <50 age group. When comparing CTS prevalence across male and female subgroups, there was no difference found between patients with and without hypothyroidism.

### 3.3. Electrodiagnostic Parameters Comparison Between Patients with and Without Hypothyroidism (Table 3 and Table 4)

Among both groups of patients with and without CTS, there were no significant differences in median sensory latency, motor latency, Palmdiff, Ringdiff, or Thumbdiff between patients with and without hypothyroidism. Similarly, comparison across all age categories did not reveal any differences. Moreover, there were no differences in electrodiagnostic parameters between patients with or without hypothyroidism among females. Males showed more prolonged latency measures among CTS patients when comparing patients with and without hypothyroidism.

## 4. Discussion

Our study evaluated the association between hypothyroidism and carpal tunnel syndrome (CTS), with a particular focus on CTS prevalence and electrodiagnostic characteristics among patients with and without hypothyroidism. Notably, our study excluded patients with DM and adjusted the analyses for age and gender, which had not been undertaken in previous studies. We excluded patients with DM as it increases the risk of CTS and significantly influences the electrodiagnostic parameter used to diagnose CTS [10,19,20]. The primary finding was a statistically significantly higher crude prevalence of CTS among patients with hypothyroidism (79.7%) compared to those without (61.8%). However, after adjusting for age and gender, the strength of this association was attenuated and lost its statistical significance. When examining age subgroups, the adjusted OR indicated a higher prevalence of CTS among patients with hypothyroidism under the age of 50 years. This could be attributed to the presence of additional risk factors that increase the risk of CTS in individuals over 50 years, such as occupation strain and age-related nerve changes [21]. Our observation aligns with the findings of Shiri et al. [1], who demonstrated in a meta-analysis that patients with hypothyroidism are approximately twice as likely to develop CTS. The authors found a stronger association between hypothyroidism and the need for CTS decompression surgery, which may suggest that conservative treatment is less effective in this population. This latter finding was also reported in recent study [22]. One possible explanation is that CTS patients with hypothyroidism are more likely to report functional limitations due to CTS symptoms [23].

The impact of hypothyroidism on electrodiagnostic latency parameters appeared to be limited. For patients without CTS, sensory and motor latencies did not significantly differ between patients with and without hypothyroidism, suggesting that subclinical nerve conduction changes may not be present in individuals with hypothyroidism but without CTS. This contrasts with prior findings by Jaiswal et al., who observed prolonged median sensory latencies in 60 patients with subclinical hypothyroidism compared to 60 healthy controls [24]. This discrepancy may be due to differences in study design: Jaiswal et al. selected healthy volunteers as controls, whereas our study included a broader clinical population referred for neurophysiological evaluation of hand symptoms, potentially representing more real-world variability. Additionally, the sensory latency values among patients with hypothyroidism in Jaiswal et al.’s study, despite statistical differences when compared with healthy controls, remained within normal limits.

For patients with CTS, sensory and motor latencies were not significantly different between those with and without hypothyroidism. This is consistent with earlier findings indicating that CTS patients with hypothyroidism often exhibit normal electrodiagnostic latencies, despite symptomatic disease [3,25]. One possible explanation is that once structural compression of the median nerve occurs, the contribution of systemic metabolic conditions such as hypothyroidism may be overshadowed by the mechanical insult. Another explanation is that hypothyroidism may reduce the nerve’s tolerance for compression, resulting in CTS symptoms manifesting at a lower threshold. In other words, even mild compression may produce symptoms in hypothyroid patients due to increased nerve susceptibility [3]. These observations suggest that, once CTS is clinically established, the electrodiagnostic profile is largely similar between patients with and without hypothyroidism. These results highlight the importance of considering thyroid disease in CTS risk assessment but not necessarily in interpreting nerve conduction study severity. Prior research also indicates that hypothyroidism does not have an impact on the severity of carpal tunnel syndrome, reinforcing the idea that hypothyroidism may play a greater role in disease initiation than in disease progression [25,26].

The findings of our study have a number of clinical implications. First, clinicians should maintain a high index of suspicion for CTS in patients with hypothyroidism, even if electrodiagnostic findings are equivocal. Second, the minimal impact of hypothyroidism on electrodiagnostic parameters in non-CTS patients suggests that routine nerve conduction screening may not be warranted in the absence of CTS symptoms. Third, the electrodiagnostic features of CTS do not differ between patients with and without hypothyroidism; hence, the standard electrodiagnostic criteria to confirm CTS should also be used in patients with hypothyroidism to confirm CTS diagnosis [27]. Confirming CTS diagnosis with electrodiagnostic studies remains an important measure to reduce the likelihood of CTS release surgery being of no benefit [18]. Finally, age stratification should be carefully considered when interpreting median nerve conduction findings in patients with hypothyroidism.

The limitations of our study include its retrospective design. Additionally, the diagnosis of hypothyroidism relied on clinical documentation and medication history rather than quantitative thyroid hormone levels, which also limits our ability to assess the relationship between hormone levels and CTS severity or nerve conduction abnormalities. Although we excluded patients with diabetes mellitus to minimize confounding, we did not control for other potential contributors such as body mass index (BMI), occupational hand use, or physical activity levels, which may influence both CTS risk and electrophysiological findings. While we stratified analyses by age and sex, the sample sizes in certain subgroups—particularly males with CTS and hypothyroidism—were relatively small, possibly limiting the statistical power of our study in detecting subtle differences. Additionally, our study did not utilize validated patient-reported outcome measures to assess symptom severity. Moreover, we did not explore the efficacy of conservative treatment versus surgical intervention and their association with the electrodiagnostic parameters in patients with hypothyroidism. Finally, the generalizability of the results of this study could be limited due to the limited number of centers included.

In conclusion, hypothyroidism may increase the risk of CTS, particularly in younger patients, but it does not significantly alter its electrodiagnostic features or severity. These findings suggest that hypothyroidism may serve as a contributing or permissive factor rather than as a determinant of disease progression. Future studies may explore the predictive value of electrodiagnostic parameters for the efficacy of conservative versus surgical interventions and may incorporate imaging modalities and longitudinal follow up to determine the outcome of CTS among patients with hypothyroidism.

## Figures and Tables

**Table 1:** Patient characteristics.

	Without CTS			With CTS		
Variable	No Hypothyroidism n = 157	With Hypothyroidism n = 14	*p*-Value	No Hypothyroidism n = 254	With Hypothyroidism n = 55	*p*-Value
Age (years), median (IQR)	41 (33–52)	41.5 (35–52)	0.841	49 (40–56)	48 (39–56)	0.773
Male, n (%)	80 (50.9%)	2 (14.3%)	0.008	44 (17.3%)	5 (9.1%)	0.130
Disease duration (months), median (IQR)	12 (2–36)	24 (2–72)	0.299	12 (4–36)	12 (5–36)	0.394
Age <30 years, n (%)	30 (19.1%)	2 (14.3%)	0.399	20 (7.9%)	2 (3.6%)	0.693
Age 30–39 years, n (%)	41 (26.1%)	3 (21.4%)		43 (16.9%)	12 (21.8%)	
Age 40–49 years, n (%)	40 (25.5%)	4 (28.6%)		70 (27.6%)	14 (25.5%)	
Age 50–59 years, n (%)	28 (17.8%)	5 (35.7%)		87 (34.3%)	21 (38.2%)	
Age ≥60 years, n (%)	18 (11.5%)	0 (0.0%)		34 (13.4%)	6 (10.9%)	
	Padua CTS severity classification					
0: Normal, n (%)	56 (35.7%)	6 (42.9%)	0.832	27 (10.6%)	5 (9.1%)	0.826
1: Minimal, n (%)	20 (12.7%)	1 (7.1%)		17 (6.7%)	5 (9.1%)	
2: Mild, n (%)	71 (45.2%)	7 (50.0%)		83 (32.7%)	16 (29.1%)	
3: Moderate, n (%)	9 (5.7%)	0 (0.0%)		110 (43.3%)	25 (45.5%)	
4: Severe, n (%)	1 (0.6%)	0 (0.0%)		16 (6.3%)	3 (5.5%)	
5: Extreme, n (%)				1 (0.4%)	1 (1.8%)	
	Bland CTS severity classification					
0: Normal, n (%)	110 (70.1%)	13 (92.9%)	0.462	83 (32.7%)	14 (25.5%)	0.497
1: Very mild, n (%)	22 (14.0%)	1 (7.1%)		34 (13.4%)	12 (21.8%)	
2: Mild, n (%)	22 (14.0%)	0 (0.0%)		62 (24.4%)	11 (20.0%)	
3: Moderate, n (%)	2 (1.3%)	0 (0.0%)		58 (22.8%)	14 (25.5%)	
4: Severe, n (%)	1 (0.6%)	0 (0.0%)		7 (2.8%)	2 (3.6%)	
5: Very severe, n (%)				9 (3.5%)	1 (1.8%)	
6: Extremely severe, n (%)				1 (0.4%)	1 (1.8%)	

IQR: interquartile range

**Table 2:** Prevalence of CTS.

	No Hypothyroidism n = 411	With Hypothyroidism n = 69	Odds Ratio (95% CI)	*p* Value
CTS, n (%)	254 (61.8)	55 (79.7)	2.42 (1.31–4.51)	0.005
CTS adjusted prevalence for age and gender, (%)	63.1	72.9	1.71 (0.89–3.28)	0.106
CTS among female, n (%)	210 (73.2)	50 (80.7)	1.52 (0.77–3.02)	0.223
CTS among male, n (%)	44 (35.5)	5 (71.4)	4.54 (0.85–24.40)	0.077
CTS among age < 50 years, n (%)	133 (54.5)	28 (75.7)	2.59 (1.17–5.73)	0.018
CTS among age 50–59 years, n (%)	87 (75.7)	21 (8087)	1.35 (0.46–3.91)	0.579
CTS among age ≥60 years, n (%)	34 (65.4)	6 (100)		0.083

**Table 3:** The electrodiagnostic parameters for the whole cohort and stratified by age.

	Without CTS			With CTS		
Variable	No Hypothyroidism n = 157	With Hypothyroidism n = 14	*p*-Value	No Hypothyroidism n = 253	With Hypothyroidism n = 54	*p*-Value
Median Sensory Latency (ms), (Median [IQR])	3.1 (2.9–3.3)	3.1 (3–3.2)	0.659	3.7 (3.3–4.5)	3.7 (3.2–4.6)	0.951
Median Motor Latency (ms), (Median [IQR])	3.3 (3–3.5)	3.2 (3.1–3.3)	0.404	3.9 (3.4–5.1)	4.0 (3.4–5.2)	1.000
Palmdiff (ms), (Median [IQR])	0.1 (0–0.3)	0.2 (0.1–0.3)	0.271	0.6 (0.3–1.1)	0.6 (0.2–1.4)	0.879
Ringdiff (ms), (Median [IQR])	0.1 (0–0.3)	0.1 (0–0.2)	0.663	0.6 (0.3–1.3)	0.6 (0.1–1.9)	0.800
Thumbdiff (ms), (Median [IQR])	0.3 (0.2–0.5)	0.3 (0.1–0.4)	0.202	0.9 (0.6–1.5)	1.0 (0.6–2.0)	0.501
	According to age group					
	without CTS			with CTS		
Variable	No hypothyroidism	With hypothyroidism	*p*-value	No hypothyroidism	With hypothyroidism	*p*-value
	Age < 50					
	n = 111	n = 9		n = 133	n = 28	
Median Sensory Latency (ms), (Median [IQR])	3.0 (2.9–3.2)	3.1 (2.9–3.1)	0.616	3.58 (3.1–4.2)	3.4 (3.1–4.3)	0.987
Median Motor Latency (ms), (Median [IQR])	3.2 (3.0–3.4)	3.2 (3.1–3.3)	0.932	3.8 (3.2–4.7)	3.9 (3.1–4.7)	0.836
Palmdiff (ms), (Median [IQR])	0.1 (0.0–0.2)	0.2 (0.05–0.3)	0.241	0.5 (0.2–1.0)	0.4 (0.1–1.3)	0.664
Ringdiff (ms), (Median [IQR])	0.1 (0.0–0.2)	0.1 (0.0–0.2)	0.831	0.5 (0.2–1.3)	0.5 (0.1–1.8)	0.976
Thumbdiff (ms), (Median [IQR])	0.3 (0.2–0.5)	0.15 (0.1–0.4)	0.113	0.8 (0.5–1.4)	0.9 (0.5–1.9)	0.762
	Age 50–59					
	n = 28	n = 5		n = 87	n = 21	
Median Sensory Latency (ms), (Median [IQR])	3.2 (3.05–3.5)	3.2 (3.1–3.2)	0.878	3.9 (3.4–5.2)	3.8 (3.4–5.1)	0.932
Median Motor Latency (ms), (Median [IQR])	3.3 (3.1–3.7)	3.1 (3.0–3.3)	0.198	4.1 (3.4–5.9)	4.0 (3.7–5.5)	0.858
Palmdiff (ms), (Median [IQR])	0.2 (0.0–0.35)	0.2 (0.1–0.2)	0.858	0.6 (0.4–1.2)	0.7 (0.4–1.7)	0.605
Ringdiff (ms), (Median [IQR])	0.1 (0.0–0.5)	0.0 (0.0–0.2)	0.221	0.7 (0.3–1.5)	1.0 (0.3–2.1)	0.558
Thumbdiff (ms), (Median [IQR])	0.4 (0.25–0.55)	0.3 (0.3–0.4)	0.919	0.9 (0.6–1.5)	1.1 (0.6–2.0)	0.386
	Age ≥ 60					
	n = 18			n = 33	n = 5	
Median Sensory Latency (ms), (Median [IQR])	3.3 (3.1–3.5)	—	NA	4.4 (3.7–4.6)	4.6 (3.5–6.3)	0.577
Median Motor Latency (ms), (Median [IQR])	3.5 (3.2–3.6)	—	NA	4.7 (4.2–5.9)	6.1 (5.2–6.4)	0.387
Palmdiff (ms), (Median [IQR])	0.3 (0.1–0.3)	—	NA	0.8 (0.6–1.4)	1.1 (0.2–1.8)	0.840
Ringdiff (ms), (Median [IQR])	0.2 (0.0–0.4)	—	NA	0.85 (0.65–1.3)	1.2 (0.1–2.3)	0.819
Thumbdiff (ms), (Median [IQR])	0.4 (0.3–0.8)	—	NA	1.1 (0.9–1.8)	1.65 (0.4–2.9)	1.000

Palmdiff: median versus ulnar latency difference through mixed palmar study; Ringdiff: median versus ulnar latency difference through digit IV/ring finger study; Thumbdiff: median versus radial latency difference through digit I/thumb study; ms: milliseconds; IQR: interquartile range.

**Table 4:** The electrodiagnostic parameters stratified by gender.

	Without CTS			With CTS		
	**Female**					
**Parameter**	**No Hypothyroidism n = 77**	**With Hypothyroidism n = 12**	***p* Value**	**No Hypothyroidism n = 209**	**With Hypothyroidism n =49**	***p* Value**
Median Sensory Latency (ms), (Median [IQR])	3.1 (2.9–3.3)	3.1 (2.95–3.2)	0.859	3.7 (3.3–4.5)	3.5 (3.2–4.4)	0.560
Median Motor Latency (ms), (Median [IQR])	3.3 (3.0–3.4)	3.1 (3.05–3.3)	0.333	4.0 (3.4–5.2)	3.9 (3.3–4.8)	0.482
Palmdiff (ms), (Median [IQR])	0.1 (0.0–0.3)	0.2 (0.1–0.3)	0.425	0.6 (0.3–1.1)	0.5 (0.2–1.3)	0.340
Ringdiff (ms), (Median [IQR])	0.1 (0.0–0.3)	0.1 (0.0–0.2)	0.589	0.7 (0.2–1.4)	0.5 (0.1–1.7)	0.583
Thumbdiff (ms), (Median [IQR])	0.3 (0.2–0.5)	0.3 (0.1–0.35)	0.172	0.9 (0.6–1.5)	0.9 (0.6–1.8)	0.813
	**Male**					
**Parameter**	**No Hypothyroidism n = 80**	**With Hypothyroidism n = 2**	***p* value**	**No Hypothyroidism n = 44**	**With Hypothyroidism n = 5**	***p* value**
Median Sensory Latency (ms), (Median [IQR])	3.2 (2.9–3.4)	3.1 (3.1–3.1)	0.809	3.7 (3.4–4.6)	5.2 (4.8–5.3)	0.044
Median Motor Latency (ms), (Median [IQR])	3.2 (3.0–3.5)	3.3 (3.2–3.4)	0.833	3.9 (3.55–4.8)	6.4 (4.6–6.9)	0.016
Palmdiff (ms), (Median [IQR])	0.1 (0.0–0.3)	0.3 (0.1–0.4)	0.372	0.5 (0.4–1.1)	1.4 (1.4–1.7)	0.016
Ringdiff (ms), (Median [IQR])	0.0 (0.0–0.2)	0.0 (0.0–0.0)	0.369	0.5 (0.3–0.9)	2.3 (1.9–2.6)	0.019
Thumbdiff (ms), (Median [IQR])	0.4 (0.2–0.6)	0.9 (0.9–0.9)	0.161	0.9 (0.5–1.4)	2.0 (2.0–2.5)	0.004

Palmdiff: median versus ulnar latency difference through mixed palmar study; Ringdiff: median versus ulnar latency difference through digit IV/ring finger study; Thumbdiff: median versus radial latency difference through digit I/thumb study; ms: milliseconds; IQR: interquartile range.

## Data Availability

All data are available upon direct request to the corresponding author.

## Abbreviations

The following abbreviations are used in this manuscript:

CTS	Carpal tunnel syndrome
OR	Odds ratio
Palmdiff	Median versus ulnar latency difference through mixed palmar study
Ringdiff	Median versus ulnar latency difference through digit IV/ring finger study
Thumbdiff	Median versus radial latency difference through digit I/thumb study
